# Association between anxiety and clinical outcomes in Chinese patients with myocardial infarction in the absence of obstructive coronary artery disease

**DOI:** 10.1002/clc.23386

**Published:** 2020-06-09

**Authors:** Chao‐Jie He, Chun‐Yan Zhu, Bin Han, Hai‐Zhen Hu, Shi‐Jun Wang, Chang‐Lin Zhai, Hui‐Lin Hu

**Affiliations:** ^1^ Department of Cardiology, The First Hospital of Jiaxing The Affiliated Hospital of Jiaxing University Jiaxing China; ^2^ Department of Psychology, The First Hospital of Jiaxing The Affiliated Hospital of Jiaxing University Jiaxing China; ^3^ Department of Nephrology, The First Hospital of Jiaxing The Affiliated Hospital of Jiaxing University Jiaxing China

**Keywords:** anxiety, myocardial infarction, outcomes

## Abstract

**Background:**

Myocardial infarction in the absence of obstructive coronary artery disease (MINOCA) accounts for approximately 5% ‐ 6% of acute myocardial infarction (AMI) patients. Anxiety symptoms are common in patients with coronary artery disease (CAD), and are associated with a poor prognosis. However, the association between anxiety and MINOCA outcomes is less clear.

**Hypothesis:**

Anxiety will be associated with clinical outcomes in patients with MINOCA.

**Methods and Results:**

Between November 2014 and December 2016, 620 hospitalized patients with MINOCA were recruited from a single center. Within 7 days of coronary angiography, anxiety was assessed using the Zung Self‐Rating Anxiety Scale. The primary endpoint was all‐cause mortality; secondary endpoint was any major adverse cardiovascular event (MACE). After 3 years, 87 deaths and 151 MACE had occurred. Kaplan‐Meier curves indicated the unadjusted rates of all‐cause mortality (log‐rank *P* = .045) and MACE (log‐rank *P* = .023) were significantly higher in the anxiety group compared with the control group of patients without anxiety. Multivariate Cox regression analysis showed that clinically significant anxiety was an independent prognostic factor for all‐cause mortality as well as MACE (hazard ratio [HR] = 1.547; 95% confidence interval [CI], 1.006‐2.380; *P* = .047; HR = 1.460; 95% CI, 1.049‐2.031; *P* = .025; respectively).

**Conclusions:**

Anxiety is significantly and independently associated with an increased risk of all‐cause mortality and MACE in patients with MINOCA.

Myocardial infarction in the absence of obstructive coronary artery disease (MINOCA) is a group of syndromes with multiple potential mechanisms characterized by evidence of myocardial infarction in the absence of obstructive (≥50%) coronary stenosis on angiography. It accounts for approximately 5% to 6% of all acute myocardial infarction (AMI) patients and has gained increasing awareness among physicians.^1^, ^2^, ^3^ The underlying pathological mechanisms of MINOCA include plaque disruption, spontaneous coronary artery dissection, coronary microvascular dysfunction, Takotsubo cardiomyopathy, myocarditis, coronary spasm, and supply‐demand mismatch.^4^, ^5^

Anxiety and depression are the most commonly studied psychological syndromes in cardiac patients, and accumulating evidence has demonstrated that anxiety and depression are associated with an increased risk of mortality and cardiac events.^6^, ^7^ The American Heart Association (AHA) considers depression a risk factor with poor prognosis among patients with acute coronary syndrome (ACS).^8^ Previously, the authors have shown that the diagnosis of depression is significantly associated with increased risk of mortality and cardiovascular events in patients with MINOCA.^9^

Anxiety is defined as a psychological and physiological state characterized by feelings of worry, uneasiness, or fear strong enough to interfere with daily life and work, which differs from depression.^10^, ^11^ Anxiety is the most common negative emotion among patients with coronary heart disease (CAD), with prevalence rates ranging from 24% to 31%.^12^ However, the impact of anxiety symptoms on the survival of patients with MINOCA is unclear. Few studies on the prevalence of anxiety in patients with MINOCA exist.^13^ In this study, we performed a prospective cohort study to determine the prevalence of anxiety and relationship between anxiety and clinical outcomes in individuals with MINOCA.

## 1 PARTICIPANTS

Our study followed the principles of the Declaration of Helsinki and was approved by the Ethics Committee at Jiaxing First Hospital, Jiaxing University, Jiaxing, China. All participants enrolled in the study signed their consent after being fully informed of the purpose of research.

Patients were recruited consecutively from November 2014 to December 2016 at a Chest Pain Center of the First Affiliated Hospital of Jiaxing University. All patients underwent emergency coronary angiography in the absence of obstructive (≥ 50%) coronary stenosis. The Chest Pain Center is located in Jiaxing, a historic city consisted of five counties and two regions, which serves six million inhabitants. Inclusion criteria were as follows: (1) patients with MINOCA; (2) agreement to participate the study. The initial diagnosis of MINOCA required the presence of (1) AMI meeting the Third Universal Definition of Myocardial Infarction criteria;^14^ (2) no artery stenosis ≥50% after angiography; (2) no other clinically overt cause for the specific presentation.^5^ The exclusion criteria were particularly strict in this study: (1) The diagnosis of MINOCA was approximately consistent with clinical algorithm recommended by AHA,^15^ and excluded patients with Takotsubo syndrome, pulmonary embolism, overlooked obstructive CAD, coronary thrombus, and myocarditis; (2) patients refused to participate in the trial; (3) cognitive dysfunction or mental confusion; (4) patients with cancer or physical disability. We have tried our best to identify the underlying causes responsible for MINOCA. If patients were suspected with myocarditis, such as a history of intestinal or respiratory infections, cardiac magnetic resonance imaging (CMRI) was recommended. If patients were suspected with coronary vasospasm, provocative testing was recommended. The MINOCA individuals were split into two groups based on anxiety diagnosis, namely, anxiety group and no anxiety group.

## 2 CLINICAL CHARACTERISTICS

Patient clinical data were acquired from the electronic patient records system using HaiTai software (version 3.0). The following demographic characteristics were collected: risk factors; laboratory, laboratory, electrocardiography, ultrasound and angiography findings; and discharge medications. Data collectors and analysts were blind to the study group assignment (anxiety or no anxiety), but the investigators were not.

## 3 ASSESSMENT OF ANXIETY

Within 1 week following coronary angiography, the Zung Self‐Rating Anxiety Scale (SAS) was administered by psychologists who were blinded to the patient' medical condition. The structured face‐to‐face interview, which takes 15 to 20 minutes to complete, was performed during hospitalization. The Zung SAS is a self‐report questionnaire comprising 20 items covering psychological, affective, and somatic symptoms that describe anxiety symptoms.^16^ This instrument was widely accepted to identify and quantify anxiety in medical patients with established reliability and validity.^17^, ^18^ For the assessment of anxiety symptoms, patients were required to describe how frequently they suffered from each symptom (such as “Have you ever felt afraid;” “Do you find yourself restless and can't sit still;” and “Do you ever feel nervous and anxious”) over the past week on a 4‐point scale ranging from “none or some of the time” to “most or all of the time.” The Cronbach α for anxiety scale was .85. The original score was calculated by adding the rated responses obtained for each of the 20 items. The standard score was calculated by the original score multiplied by 1.25, which ranged from 25 to 100 points. SAS standard scores of 50 or higher indicate the presence of clinically significant anxiety.^16^

## 4 PRIMARY AND SECONDARY END POINTS

All participants were followed up for 3 years from anxiety ascertainment. Patients were contacted by telephone or instant messaging (WeChat) each month. We defined all‐cause mortality as the primary endpoint and major adverse cardiovascular events (MACE) as the secondary endpoint. MACE was defined as the occurrence of cardiovascular death, coronary artery bypass grafting (CABG), heart failure, stroke, and cardiovascular‐related rehospitalization.^19^ Cardiovascular‐related rehospitalization, was defined as readmission in the presence of angina with positive troponin or received percutaneous coronary intervention. The confirmation of endpoints was performed by two senior cardiologists who were blinded to admission anxiety status. Death was confirmed by verification of death certificates.

## 5 STATISTICAL ANALYSIS

Statistical analysis was performed using IBM SPSS‐statistics 22.0 (IBM, SPSS Inc., Chicago, IL). Continuous variables were compared by Student's *t* test and categorical variables by Chi‐square test or Fisher's exact test. Demographic and laboratory data are presented as mean ± SD. The cumulative incidence of all‐cause mortality and MACE in both groups was estimated according to the Kaplan‐Meier curves using the log‐rank test statistic. Multiple Cox regression models were used to examine associations between anxiety and clinical outcomes by comparing the two groups. In order to address the predictive value of anxiety for all‐cause mortality or MACE, we simultaneously adjusted for the following covariates: age, sex, traditional cardiovascular risk factors, medications at discharge, and electrocardiography changes. The proportional hazards assumption was evaluated for Cox regression. Results were estimated as hazard ratios (HR) with 95% confidence intervals (95% CI). An alpha level of .05 or less was considered as significant for all statistical procedures.

## 6 RESULTS

### 6.1 Patient characteristics stratified by anxiety

Between November 2014 and December 2016, 6750 patients were diagnosed with myocardial infarction from the Chest Pain Center. In total, 694 patients with MINOCA at admission were screened. Of that total, 74 individuals were excluded from the current study: 18 with Takotsubo syndrome, 33 with myocarditis, 4 with pulmonary embolism, 4 with coronary thrombus, 2 with overlooked obstructive CAD, and 15 who declined to participate in the study. Among the 620 participants who enrolled in the study, 605 patients were successfully followed up, 4 patients dropped out, and 11 patients were lost to follow up. The baseline characteristics of the patients are presented in Table 1. Among the 620 participants, the mean age was 63.7 ± 12.9 years, the majority (360; 58.1%) were female, 139 (22.4%) had a history of hypertension and 131 (21.2%) had a history of diabetes. Of the 620 patients who enrolled in the study, 186 participants had SAS scores greater than or equal to 50 (anxiety group), and 434 were not (control group). The incidence of anxiety symptoms was 30% in Chinese patients with MINOCA. The group with clinical anxiety had a higher proportion of female patients (*P* < .05) compared with the control group; there were no significant differences between the groups for other demographic and laboratory characteristics. A final diagnosis for MINOCA group was found in 183 (29.5%). The final diagnoses were coronary vasospasm 47 (25.7%), plaque disruption 28 (15.3%), CMRI‐confirmed MINOCA 96 (52.5%), coronary embolism 7 (3.8%), and coronary dissection 5 (2.7%).

**TABLE 1:** Baseline characteristics of the study population with or without anxiety symptoms

Characteristics	With anxiety (n = 186)	Without anxiety (n = 434)	*P* value
Demographics			
Age, mean ± SD (y)	63.6 ± 12.9	63.8 ± 12.5	.759
Female, n (%)	119 (64.0)	241 (55.5)	.031
BMI, mean ± SD (kg/m^2^)	24.2 ± 3.6	24.6 ± 3.6	.667
Risk factors, n (%)			
Smoking	44 (23.7)	95 (21.9)	.167
Hypertension	90 (48.4)	216 (49.8)	.767
Diabetes	41 (22.0)	90 (20.7)	.556
Hyperlipemia	49 (26.3)	109 (25.1)	.799
Stroke history	7 (3.8)	15 (3.5)	.889
Heart failure	6 (3.2)	16 (3.7)	.826
Medications at discharge, n (%)			
Anti‐platelets	149 (80.1)	357 (82.3)	.524
βBlockers	112 (60.2)	254 (58.5)	.498
RAAS inhibitors	92 (49.4)	203 (46.7)	.110
Statins	152 (81.7)	360 (82.9)	.764
Electrocardiographic changes on admission, n (%)			
STEMI	24 (12.9)	55 (12.6)	.712
NSTEMI	162 (87.1)	379 (87.4)	.881
Echocardiography, mean ± SD			
LVEF (%)	54.4 ± 13.2	54.8 ± 13.5	.815
Laboratory parameters on admission, mean ± SD			
Pro‐BNP (pg/mL)	912.4 ± 1320.6	929 ± 1296.3	.778
cTnT (ng/mL)	1.9 ± 1.0	2.0 ± 0.9	.795
CRP (mg/L)	15.9 ± 4.9	16.7 ± 5.3	.844
Angiographic data, n (%)			
Normal vessels	18 (9.7)	40 (9.2)	.871
Stenosis≤30%	90 (48.4)	202 (46.5)	.256
30%<Stenosis<50%	78 (41.9)	192 (44.2)	.182

Abbreviations: BMI indicates body mass index; cTnT, cardiac troponin T; CRP, C reactive protein; LVEF; left ventricular ejection fraction; NSTEMI, non‐ST‐segment elevation myocardial infarction; Pro‐BNP, pro‐brain natriuretic peptide; RAAS, rennin‐angiotensin‐aldosterone system; STEMI, ST‐segment elevation myocardial infarction.

### 6.2 Association of anxiety with clinical outcomes

**FIGURE 1:**
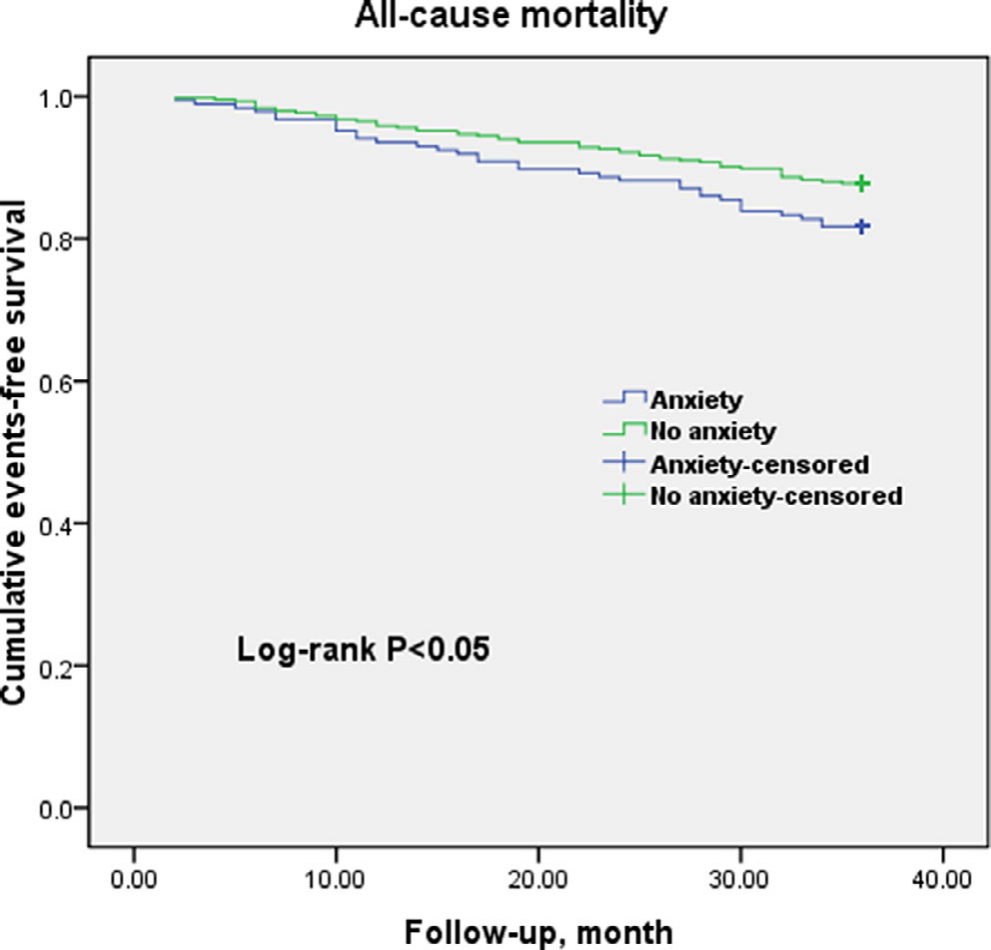
Cumulative events‐free survival (Kaplan‐Meier curves) for all‐cause mortality in MINOCA patients with or without anxiety

**FIGURE 2:**
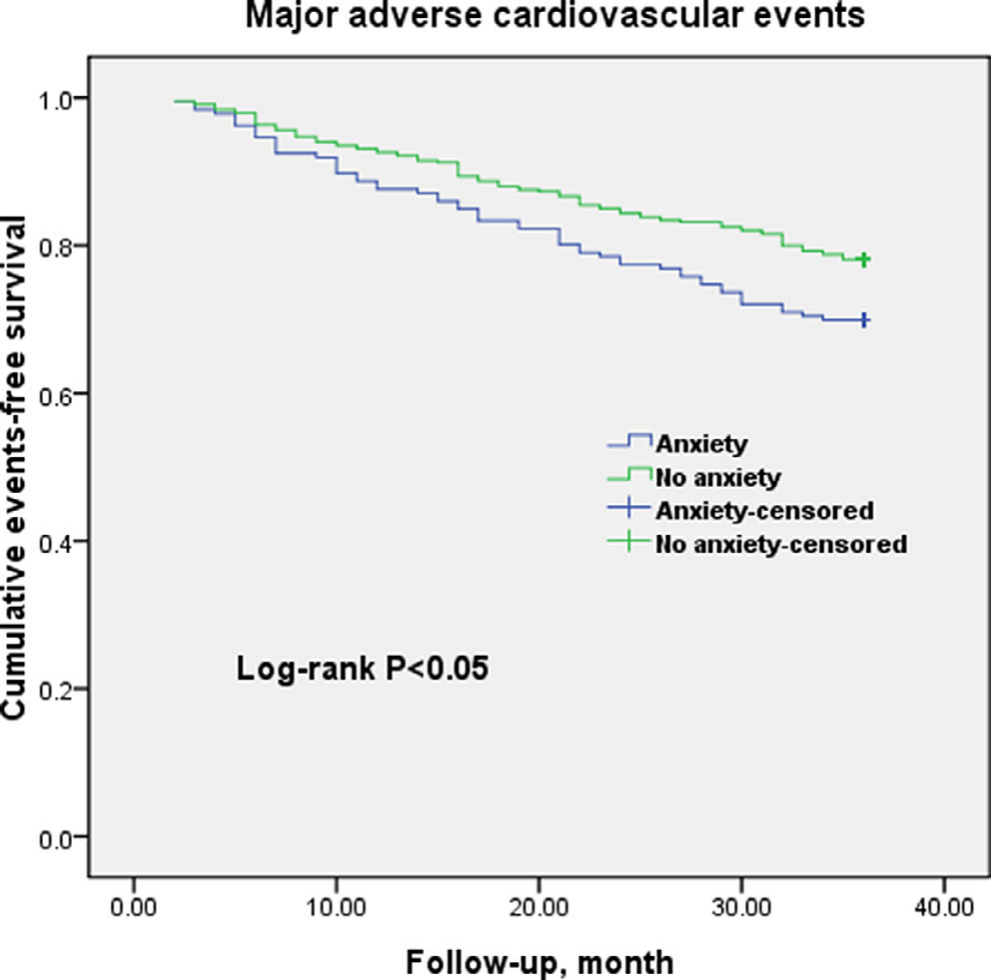
Cumulative events‐free survival (Kaplan‐Meier curves) for MACE in MINOCA patients with or without anxiety

At the 3‐year follow up, a total of 87 deaths and 151 MACE had occurred. Of these totals, in patients with anxiety, there were 34 deaths and 56 MACE (32 cardiovascular deaths, 0 CABG, 3 heart failures, 1 stroke, and 21 cardiovascular‐related rehospitalizations; in the control group, there were 53 deaths and 95 MACE (49 cardiovascular deaths, 1 CABG, 4 heart failures, 2 strokes, and 39 cardiovascular‐related rehospitalizations). Figures 1 and 2 show Kaplan‐Meier curves, which indicate that the unadjusted rates of all‐cause mortality (log‐rank *P* = .045) and MACE (log‐rank *P* = .023) were significantly higher in patients with clinically significant anxiety compared to the group without anxiety. We used a simple method that to plot the data and see if the survival curves cross for checking the proportional hazards assumption before running a Cox regression. The proportional hazards assumption was satisfied in this study. Univariate Cox regression analysis showed that anxiety was the independent prognostic factor for all‐cause mortality (HR = 1.678; 95% CI, 1.056‐2.673; *P* = .034) and MACE (HR = 1.613; 95% CI, 1.117‐2.634; *P* = .011) in the MINOCA population. After adjusting for traditional cardiac risk and potential protective medications in multivariate analysis, anxiety remained an independent prognostic factor for all‐cause mortality as well as MACE (HR = 1.547; 95% CI, 1.006‐2.380; *P* = .047; HR = 1.460; 95% CI, 1.049‐2.031; *P* = .025; respectively, Tables 2 and 3). Notably, renin‐angiotensin‐aldosterone system (RAAS) inhibitors, statins, and ST‐segment elevation myocardial infarction (STEMI) showed a significant association with clinical outcomes, consistent with the findings of previous research.

**TABLE 2:** Multivariate Cox regression of variables influencing all‐cause mortality

Variables	HR (95% CI)	*P* value
Age	1.011 (0.965‐1.067)	.656
Female	0.851 (0.602‐1.125)	.268
Smoking	1.274 (0.835‐1.542)	.119
Hypertension	1.102 (0.796‐1.345)	.752
Diabetes	1.156 (0.842‐1.386)	.446
Heart failure	1.351 (0.890‐1.871)	.094
Anti‐platelets	0.896 (0.742‐1.210)	.654
β‐Blockers	0.753 (0.494‐1.213)	.242
RAAS inhibitors	0.689 (0.425‐0.986)	.039
Statins	0.411 (0.265‐0.612)	<.001
STEMI	5.223 (2.192‐14.225)	<.001
Anxiety	1.547 (1.006–2.380)	.047

Abbreviations: HR indicates hazard ratio; RAAS, rennin‐angiotensin‐aldosterone system; STEMI, ST‐segment elevation myocardial infarction.

**TABLE 3:** Multivariate Cox regression of variables influencing MACE

Variables	HR (95% CI)	*P* value
Age	1.026 (0.946‐1.089)	.823
Female	0.906 (0.772‐1.165)	.364
Smoking	1.121 (0.889‐1.342)	.771
Hypertension	1.174 (0.856‐1.603)	.561
Diabetes	1.114 (0.902‐1.553)	.449
Heart failure	1.396 (0.896‐2.064)	.102
Anti‐platelets	0.821 (0.651‐1.136)	.339
β‐Blockers	0.653 (0.522‐1.241)	.251
RAAS inhibitors	0.615 (0.421‐0.912)	.007
Statins	0.402 (0.289‐0.576)	<.001
STEMI	7.232 (2.226‐16.537)	<.001
Anxiety	1.460 (1.049–2.031)	.025

*Note:* MACE indicates major adverse cardiovascular events.

Abbreviations: HR, hazard ratio; RAAS, rennin‐angiotensin‐aldosterone system; STEMI, ST‐segment elevation myocardial infarction.

## 7 DISCUSSION

To our knowledge, this is the first prospective cohort study to investigate the prevalence of anxiety in Chinese patients with MINOCA and its association with clinical outcomes. In our study, anxiety was common among individuals with MINOCA, with prevalence similar to that in CAD populations. Daniel et al^13^ found that anxiety and depression are common in patients with MINOCA, with prevalence rates similar to patients with coronary heart disease. The incidence of anxiety symptoms was 30% in Chinese patients with MINOCA, which is consistent with prevalence of anxiety measured by Hospital Anxiety and Depression Scale (HADS) in Daniel's work. The Zung SAS was administered by psychologists within 1 week following coronary angiography in this study, which differs from Daniel's work that HADS was administrated at the 3 month‐visit after myocardial infarction. The high prevalence of anxiety is in line with previous study that demonstrated that low quality of life scores in patients with MINOCA.^20^ In Daniel's study, Takotsubo syndrome was common in patients with MINOCA. In one retrospective study of Chinese patients with MINOCA, Takotsubo syndrome was found in 14 of 128 patients with MINCOA.^21^ In our study, 18 patients with Takotsubo syndrome were excluded from analysis. It needs further study to determine whether the prevalence of Takotsubo syndrome was lower in Pacific race. We found that anxiety was significantly and independently associated with increased risk of all‐cause mortality and MACE in patients with MINOCA. Compared to our earlier studies on depression on all‐cause mortality and cardiovascular events in this patient population, anxiety did not have as strong an association with these outcomes.^9^

Previous studies have demonstrated that anxiety can be connected with a pathological alteration in cardiac autonomic tone, with either increased sympathetic function or impaired vagal control.^22^, ^23^ Anxiety has been connected with chronically increased catecholamine levels, which has been demonstrated to activate the coagulation and fibrinolysis system in the direction of a hypercoagulable state.^24^, ^25^, ^26^ In addition, Zafar et al^24^ have shown that anxiety was associated with increased serotonin‐mediated platelet aggregation in CAD patients. Several studies have demonstrated that individuals with anxiety had longer QT intervals and larger QT dispersion.^27^, ^28^, ^29^ Finally, patients with anxiety symptoms can have unhealthy lifestyle behaviors, such as smoking, drinking, overeating, lack of exercise, poor interpersonal relationship, and low medication adherence,^30^ although we found there were no significant differences in demographic and basic characteristics between both groups in our study. All the above mechanisms and causes might be responsible for the increased risk of all‐cause mortality and MACE in this study.

Our research group has previously shown that depression is associated with increased risk of death and cardiovascular events in MINOCA patients.^9^ The current study used the Zung SAS, which was shown to be valid and reliable in both the general population and patients with comorbidities; assessment of anxiety symptoms was made by psychologists who were blinded to patients' medical conditions.^16^ Previously reported confounding factors that might influence outcomes, such as RAAS inhibitors, β‐Blockers, anti‐platelets, statins, and STEMI were adjusted for in our analyses.^31^ And of note, we used instant messaging software WeChat to conduct follow up, with only 11 patients remaining unaccounted for at the end of the 3‐year follow‐up period.

The design of current study has some strengths. First, the diagnosis process of MINOCA we adopted in this study was approximately consistent with clinical algorithm recommended by AHA,^15^ we strictly excluded clinically overt causes for a myocardial injury, such as myocarditis, Takotsubo syndrome, pulmonary embolism, and potentially overlooked obstructive CAD. Thus, the definition of MINOCA in the study is in accordance with the statement of contemporary diagnosis and management of patients with MINOCA, thereby further supporting our results. Second, the well‐known confounding factors that affect clinical outcomes in patients with MINOCA, such as traditional cardiovascular risk factors, RAAS inhibitors, STEMI, and statins, were all analyzed for adjustment.

Several potential limitations in this study were acknowledged. First, symptoms of anxiety and depression may coexist in coronary heart disease patients, and we did not adjust for depression that were reported to be correlated with anxiety disorders and predict adverse clinical outcomes in patients in MINOCA.^32^, ^33^ Although numerous studies have shown that anxiety was an independent prognostic factor of cardiovascular events in CAD patients after adjusting for depression.^34^, ^35^ Second, because we did not collect data from patients who refused consent, and because this was an observational study, the percent of patients who refused to participate in the trial was relatively low. Third, patients involved in this study were from a single Chest Pain Center, and the results may not be generalizable. Fourth, we did not assess the anxiety disorders during the 3‐year follow‐up period, and the improvement or progression of anxiety symptoms might have had an effect on clinical outcomes in patients with MINOCA. Fifth, Mayou's study of patients with myocardial infarction reported that there were improvements in mean scores for anxiety between admission and at 3 months assessment. In this study, we have assessed anxiety symptoms within 1 week following coronary angiography, which may include cases with symptoms due to the stress reactions inflicted by the acute event.^13^, ^36^

## 8 CONCLUSIONS

In conclusion, the incidence of anxiety in Chinese patients with MINOCA is high. Anxiety is significantly and independently associated with increased risk of all‐cause mortality and MACE in this group; however, this relationship is not as strong as that of co‐morbid depression. The results of the current study remind us to pay attention to this often‐neglected population. Further research is warranted to confirm the potential benefit of psychological intervention for anxiety disorders in individuals with MINOCA.

## CONFLICT OF INTEREST

The authors declare no potential conflict of interests.
